# The association between estimated pulse wave velocity and balance function in U.S. adults

**DOI:** 10.1038/s41371-025-01062-0

**Published:** 2025-09-03

**Authors:** Andrew R. Heckel, Alaina C. Glasgow, Wonhee Cho, Joon Young Kim

**Affiliations:** 1 Department of Exercise Science, Syracuse University, Syracuse, NY USA; 2 Exercise Science & Kinesiology, Juniata College, Huntingdon, PA USA

**Keywords:** Risk factors, Ageing

## Abstract

Vascular dysfunction has been shown to negatively impact physical function, with arterial stiffening being linked to worsening balance function. Estimated pulse wave velocity (ePWV), derived from age and blood pressure (BP), is an emerging method of assessing vascular function that can be widely used in large, observational studies. Whether ePWV is associated with balance function in US adults remains unknown. A total of 3276 adult men and women from the 2021–2023 National Health and Nutrition Examination Survey who completed BP and balance function measures were included in this study. Balance function was assessed using the Modified Romberg Test of Standing Balance on Firm and Compliant Support Surfaces (MRT). ePWV was used to assess vascular function and derived from a regression equation using age and mean blood pressure. A general linear model was then used to determine whether ePWV is significantly associated with the number of MRT conditions passed. The model showed that, after covarying for age, systolic BP, diastolic BP, gender, race/ethnicity, educational attainment, socioeconomic status, alcohol, smoking, body mass index, and physical activity levels, ePWV was significantly and inversely related to the number of MRT conditions passed (β = −0.398, p < 0.001). Higher ePWV is associated with worsened balance control in a nationally representative sample of US adults. As ePWV only requires age and BP, measures that are taken routinely at physical examinations, future studies should longitudinally examine associations between ePWV and balance function in a clinical setting.

## Introduction

Vascular dysfunction is a robust facilitator for disability and functional impairment in adults [1–4]. Arterial stiffness has been linked to end-target organ damage due to increased pulsatile hemodynamics [5–7], which may negatively impact physical function (i.e., gait speed, muscular power) and morbidity in adults [1, 3, 4]. Specifically, deficits in balance function is a major risk factor for falls and subsequent disability [8, 9], with failure during the Modified Romberg Test of Standing Balance on Firm and Compliant Support Surfaces (assesses balance function) associated with at least a 2x increased risk for a fall in the past year [10]. Over 14 million adults in the United States (US) report falling each year [9], with accidental falls being the leading cause of injury and injury-related deaths among older adults in the US [8]. Thus, accessible methods to determine vascular health and balance function are needed for widespread screening to reduce disability and morbidity in the US adult population.

Recent studies have supported the link between arterial stiffness and balance function. Systolic hypertension is associated with worsened postural stability in older adults [11], while higher carotid-femoral pulse wave velocity (cfPWV), a measure for central arterial stiffness, is significantly associated with worsened postural control in older adults [12]. Furthermore, as vascular remodeling begins in early adulthood [13], vascular links to balance control are not limited to older adults such that vascular contributions to worsening balance control may begin in young adulthood. Likewise, we previously showed that carotid-femoral pulse wave velocity (cfPWV) independently predicts postural sway in 112 young adults (18–35 years old) [14], indicating that arterial stiffening may influence balance control throughout adulthood. However, even with these clinically important findings, it is acknowledged that the limited number of participants and narrow age range hinder the generalization of our findings. To address these limitations, we sought to use population data from the National Health and Nutrition Examination Survey (NHANES) and assess vascular function via estimated pulse wave velocity (ePWV), derived from age and blood pressure [15], which can be widely used in large, observational studies. Whether ePWV is associated with balance function remains unknown.

Therefore, the purpose of this study is to examine whether ePWV, an emerging measure of vascular aging, is associated with balance function in a nationally representative sample of US adults from NHANES. It is hypothesized that higher ePWV will be significantly associated with worsened balance function in US adults.

## Methods

### Study design

Data for this cross-sectional study were obtained from the 2021–2023 NHANES cycle. NHANES is a nationally representative survey of the civilian, non-institutionalized US population. NHANES sample selection utilizes a complex, stratified, multistage probability design to eliminate potential selection bias. All methods utilized in the NHANES protocol were approved by the National Center for Health Statistics Research Ethics Review Board and all participants provided informed consent. All methods were performed in accordance with relevant guidelines and regulations. The data for this study and the descriptions for the study procedures are publicly available online at https://wwwn.cdc.gov/nchs/nhanes/continuousnhanes/default.aspx?Cycle=2021-2023. The 2021–2023 NHANES dataset includes 12,592 participants. Participants were excluded from this analysis if they were <18 years or ≥65 years old (*n* = 7025). Participants who did not attempt at least one trial for the Modified Romberg Test of Standing Balance on Firm and Compliant Support Surfaces, had missing blood pressure (BP) data, or had invalid physical activity data (*n* = 2291) were excluded from the final analysis. Final analyses were performed on 3276 US adult men and women. As the sample size for the present study was determined based on existing data from the 2021–2023 NHANES cycle, a priori sample size calculations were not conducted. However, post-hoc power analysis (G*Power v3.1) demonstrated that the current sample size (*n* = 3276) achieved a statistical power of >99%.

### Estimated pulse wave velocity

Brachial systolic (SBP) and diastolic blood pressure (DBP) measurements were taken using an electronic, oscillometric BP measurement device with an appropriately sized cuff in the seated position following a quiet 5-min rest (Omron HEM-907XL). Three consecutive measures were completed 60 s apart, with SBP and DBP averaged across all three measures. Mean arterial pressure (MAP) was then calculated as [DBP + ((0.4)(SBP–DBP))]. ePWV was subsequently calculated based on age and MAP using the following algorithm [16]: 9.587–(0.402*age) + (4.560*0.001*age^2^)–(2.261*0.00001*age^2^*MAP) + (3.176*0.001*MAP)–(1.832*0.01*MAP). It should be noted that while MAP is traditionally calculated as [DBP + (1/3)(SBP–DBP)], a form factor of 0.4 has been shown to be more closely association with end-target organ damage [16, 17]. As such, this method for calculating MAP is the standard for studies exploring the association of ePWV with clinical outcomes and is in agreement with the Reference Values for Arterial Stiffness Collaboration [16–21].

### Balance function

Balance function was assessed using the Modified Romberg Test of Standing Balance on Firm and Compliant Support Surfaces (MRT). Detailed descriptions for MRT procedures are provided on the NHANES website (https://wwwn.cdc.gov/Nchs/Data/Nhanes/Public/2021/DataFiles/BAX_L.htm). In summary, the MRT assesses an individual’s ability to stand unassisted in five different conditions: (1) bare floor with eyes open, (2) bare floor with eyes closed, (3) dense foam surface with eyes open, (4) dense foam surface with eyes closed, and (5) dense foam surface with eyes closed while moving head slowly from side-to-side. Participants were given two trials to pass the requisite time interval for each condition. For conditions 1 and 2, a time interval of 15 s was considered passing, and for conditions 3 through 5, a time interval of 20 s was considered passing. Participants who did not complete the time interval for the requisite condition on both trials were considered to be failed. Participants were then reported as having passed or failed the MRT condition, and pass/fail status was reported for all five conditions.

### Covariates for ePWV and balance function

Potential covariates for the relationship between ePWV and balance function included age (years), gender (male/female), race/ethnicity (Mexican American, other Hispanic, Non-Hispanic White, Non-Hispanic Black, Non-Hispanic Asian, other race/multiracial), educational attainment (highest grade or level of school completed, or the highest degree achieved), socioeconomic status (SES; ratio of family income to poverty level), alcohol consumption (average number of alcoholic beverages per day in the last year, included as a covariate due to high alcohol consumption having inverse relations with arterial stiffness and postural stability [22, 23]), smoking status (whether or not participant had smoked 100 cigarettes in their lifetime [yes/no]), body mass index (BMI; weight-to-height ratio as kg/m^2^) and self-reported leisure-time moderate-to-vigorous physical activity levels (MVPA; minutes per week) [24].

### Statistical analysis

We performed all analyses by adjusting for the complex, multistage survey sampling design of NHANES. Descriptive statistics are reported using both unweighted (mean ± standard deviation (SD) and *n*, %) and weighted (mean ± standard error (SE) and frequency, %) results. The association between ePWV and the number of MRT conditions passed was assessed using bivariate two-tailed Pearson correlation coefficient. A general linear model was then used to determine whether ePWV was significantly associated with the number of MRT conditions passed. Two general linear models were performed: (1) unadjusted and (2) adjusted for the constituent components of ePWV (age, SBP, and DBP) and additional covariates including gender, race/ethnicity, educational attainment, SES, alcohol, smoking, BMI, and MVPA. We also included an adjusted model without age, SBP, or DBP to account for potential issues of multicollinearity with ePWV. Additional general linear models stratified by gender were performed to account for potential gender differences in ePWV and balance function. All statistical analyses were performed using IBM SPSS Statistics version 28 (SPSS Inc., Chicago, IL) and statistical significance was set a priori at *p* < 0.05.

## Results

### Participant characteristics

Participant characteristics are presented in Table 1. Participants in our cohort ranged in age from 20–64 years old, with 70.7% being overweight or obese and 26.7% having hypertension. We had an approximately even split between male and female participants (male = 50.5%, female = 49.4%), with most participants being NH White (57.5%). For the MRT conditions, 0.2% of participants did not pass any conditions, 0.4% passed one condition, 0.4% passed two conditions, 12.2% passed three conditions, 29.5% (passed four conditions, and 57.3% passed all five conditions.

**Table 1:** Participant Characteristics of the Sample (n = 3276).

Characteristic	Mean ± SD; *n*, % (unweighted)	Mean ± SE; frequency, % (weighted)
Age (years)	44.5 ± 13.3	41.63 ± 0.44
Gender		
Male	1481, 45.2	78073801, 50.5
Female	1795, 54.8	76577325, 49.5
Race/Ethnicity		
Mexican American	291, 8.9	13442734, 8.7
Other Hispanic	389, 11.9	16270070, 10.5
NH White	1780, 54.3	88890796, 57.5
NH Black	397, 12.2	16350378, 10.6
NH Asian	217, 6.6	10889819, 7.0
Other race/multiracial	202, 6.2	8807329, 5.7
Educational Attainment		
<9th grade	130, 4.0	5218321, 3.4
9th–11th grade	223, 6.8	8524953, 5.5
High school diploma	654, 20.0	37168815, 24.0
Some college or AA degree	997, 30.4	45936877, 29.7
College graduate or above	1271, 38.8	57780731, 37.4
SES	3.0 ± 1.7	3.1 ± 0.11
Alcohol Consumption (average per day in last year)	3.6 ± 28.6	4.5 ± 1.25
Smoking		
≥100 cigarettes in lifetime	1196, 36.5	52299890, 33.9
<100 cigarettes in lifetime	2080, 63.4	102121691, 66.0
BMI (kg/m^2^)	29.2 ± 6.7	29.1 ± 0.29
MVPA (minutes/week)	277.9 ± 404.7	285.9 ± 11.30
Blood Pressure (mmHg)		
Systolic	118.9 ± 15.4	118.0 ± 0.42
Diastolic	75.6 ± 10.5	75.1 ± 0.36
ePWV	8.2 ± 1.6	7.9 ± 0.45

### Association between ePWV and balance function

Higher ePWV was significantly associated with a lower number of MRT conditions passed (r = −0.293, *p* < 0.001). Table 2 presents results from the general linear model evaluating the relationship between ePWV and the number of MRT conditions passed. Unadjusted analysis showed that ePWV was significantly and inversely associated with the number of MRT conditions passed, with ePWV accounting for 8.6% of the variance in the number of MRT conditions passed (Estimate = −0.146 [95% CI: −0.166–−0.126], *p* < 0.001). Adjusted analysis included age, SBP, and DBP, gender, race/ethnicity, educational attainment, SES, alcohol consumption, smoking, BMI, and MVPA. The overall fit of the model was statistically significant, with the model accounting for 15.2% of the variance in the number of MRT conditions passed. ePWV remained inversely and significantly associated with the number of MRT conditions passed (Estimate = −0.248 [95% CI: −0.385–−0.110], *p* = 0.002), while SBP, DBP, SES, educational attainment, and alcohol consumption were also significant contributors to the overall model fit (*p* < 0.05). Finally, to address potential issues of multicollinearity between ePWV and its components, we removed age, SBP, and DBP from the general linear model. The removal of these variables did not change our overall results, as the overall fit of the model remained statistically significant (R^2^ = 0.124), with ePWV being inversely and significantly associated with the number of MRT conditions passed (Estimate = −0.148 [95% CI: −0.179–−0.118], *p* < 0.001). Educational attainment, smoking, and alcohol consumption were also significant contributors to the overall model fit (*p* < 0.05). Results from adjusted analysis without age, SBP, and DBP are shown in Supplementary Table S1.

**Table 2:** Results evaluating the association between ePWV and balance function.

	Model 1				Model 2			
	Standardized β	*t*	95% CI	*p*-value	Estimate	*t*	95% CI	*p*-value
**Unadjusted Model**								
ePWV	−0.303	−18.167	−0.163–0.131	*<0.001	−0.146	−15.746	−0.166–0.126	*<0.001
**Adjusted Model**								
Age	−0.043	−0.653	−0.010–0.005	0.514	0.002	0.357	−0.010–0.014	0.726
SBP	0.124	2.689	0.002–0.011	*0.007	0.008	2.846	0.002–0.013	*0.012
DBP	0.073	2.046	0.000–0.011	*0.041	0.009	2.439	0.001–0.016	*0.028
Gender (ref. female)	0.034	1.597	−0.012–0.117	0.110	−0.045	−1.226	−0.123–0.033	0.239
Race/Ethnicity	−0.003	−0.157	−0.024–0.020	0.875				
Mexican American					0.094	1.130	−0.083–0.271	0.276
Other Hispanic					0.023	0.259	−0.169–0.216	0.799
NH White					0.042	0.534	−0.126–0.211	0.601
NH Black					0.105	1.321	−0.064–0.274	0.206
NH Asian					0.143	1.691	−0.037–0.323	0.112
Other race/multiracial (ref)					---	---	---	---
Educational Attainment	0.129	5.429	0.064–0.137	*<0.001				
<9th grade					−0.359	−2.953	−0.619–0.100	*0.010
9th–11th grade					−0.272	−2.878	−0.474–0.071	*0.011
High school diploma					−0.180	−3.168	−0.300–0.059	*0.006
Some college or AA degree					−0.151	−3.653	−0.239–0.063	*0.002
College graduate or above (ref)					---	---	---	---
SES	0.083	3.515	0.017–0.060	*<0.001	0.030	2.139	0.000–0.060	*0.049
Alcohol Consumption	−0.024	−1.222	−0.002–0.001	0.222	−0.001	−5.781	−0.001–0.001	*<0.001
Smoking ref. ( < 100 cigarettes)	0.039	1.872	−0.003–0.124	0.061	−0.186	−1.693	−0.419–0.048	0.111
BMI	0.029	1.362	−0.001–0.008	0.173	0.003	0.812	−0.005–0.011	0.429
MVPA	0.001	0.070	0.000–0.001	0.945	0.001	−0.465	0.000–0.001	0.649
ePWV	−0.398	−4.470	−0.271–0.106	*<0.001	−0.248	−3.845	−0.385–0.110	*0.002

Model 1: Unweighted analysis using linear regression to evaluate the association between ePWV and balance function.

Model 2: Weighted analysis using general linear model to evaluate the association between ePWV and balance function.

### Sex-specific analysis

We previously found that female sex was inversely related to balance function in young adults [14]; therefore, we also ran separate unadjusted and adjusted analyses for males and females. In this study, we found consistent results between males and females, in that ePWV was significantly associated with the number of MRT conditions passed in unadjusted and adjusted analyses for both sexes. In unadjusted analysis, ePWV was significantly and inversely related to the number of MRT conditions passed (males: estimate = −0.121 [95% CI: −0.166–−0.077], *p* < 0.001; females estimate = −0.169 [95% CI: −0.192–−0.146], *p* < 0.001). In adjusted analysis for age, SBP, and DBP, race/ethnicity, educational attainment, SES, alcohol consumption, smoking, BMI, and MVPA; ePWV remained inversely and significantly associated with the number of MRT conditions passed for both males and females (males: −0.350 [95% CI: −0.557–−0.143], *p* = 0.003; females: estimate = −0.199 [95% CI: −0.294–−0.105], *p* < 0.001). Results from sex-specific analysis are shown in Supplementary Table S2.

## Discussion

The present study utilized a nationally representative dataset of the US population to investigate whether vascular function is associated with balance function in US adults. To pursue our study aim, we utilized useful and reliable tools for measuring vascular aging (ePWV) and balance function (MRT), which are applicable to this population-based study [15, 16]. We found that ePWV was inversely related to the number of MRT conditions passed. In both unadjusted and adjusted models, higher ePWV was significantly associated with a lower number of MRT conditions passed, with these findings consistent in both males and females. Overall, these findings suggest that higher ePWV is associated with worsened balance control in US adult men and women, and that ePWV may provide novel insight into the relationship between vascular aging and balance function.

Findings from the present study support the emerging literature connecting vascular aging with worsened balance function. Previous research regarding the association between vascular aging and balance function has been limited to connecting balance function with clinical CVD outcomes, such as stroke and chronic heart failure [25, 26]. Balance disorders have been found to predict CVD mortality [24], while balance function is significantly impaired following CVDs and cerebrovascular diseases [25–27]. More recently, studies have begun focusing on pre-clinical markers of CVD risk to relate vascular aging to balance function in older adults. Turusheva et al. [28] found that a higher cardio-ankle vascular index, a novel measure of whole-body arterial stiffness, was related to an increased risk for a fall-related injury in older adults (OR: 3.52, 95% CI: 1.03–12.04); while Peultier-Celli et al. [12] found that higher cfPWV was associated with worsened postural control among older adults in both eyes open and eyes closed conditions (*p* < 0.05 for both conditions). Together, these findings indicate that older adults with higher arterial stiffness may have more difficulty with balance control and thus an increased likelihood for fall-related injuries. We previously [14] observed that increased Mobil-O-Graph PWV significantly predicted a larger postural sway during the eyes closed standing on a foam surface condition in young adults, even after adjustment for measures of physical fitness (standardized *β* = 0.44, *p* = 0.009). The findings from our present study further extend upon these previous findings by revealing that increased ePWV is inversely associated with the number of conditions passed on the MRT in a nationally representative sample of young- and middle-aged US adults.

Potential mechanisms for the contribution of vascular aging to worsened balance function include increases in hemodynamic pulsatility due to arterial stiffness, leading to damage to the structures governing balance function [7, 29–31]. Balance function is governed by a complex set of sensorimotor systems. Inputs from visual, proprioceptive, and vestibular systems are integrated by the central nervous system, which then sends signals to the musculoskeletal system to control motor output [31]. Arterial stiffening has been linked to end-target organ damage due to increased pulsatile hemodynamics [6], and the sensorimotor structures governing balance function could be particularly vulnerable to these consequences of arterial stiffening. Hemodynamic pulsatility due to arterial stiffening may lead to damage of the retinal arterioles, cerebrovasculature, and arteries feeding the inner ear [7, 29, 30], potentially causing injury to the visual, proprioceptive, and vestibular structures responsible for balance control, leading to a worsening of balance function. Indeed, cross-sectional studies have found that higher arterial stiffness is associated with damage to the aforementioned sensorimotor structures [7, 29, 30], even in young- to middle-aged adults [29], supporting these potential mechanisms connecting arterial stiffness to balance function.

Furthermore, the findings from the present study suggest that the clinical utility of ePWV extends beyond CVD outcomes, providing valuable insight into physical functioning. ePWV is a well-established independent predictor for all-cause mortality and clinical CVD outcomes [16, 19, 20, 32], with improvements in ePWV via antihypertensive interventions even predicting lower risk for all-cause mortality in the Systolic Blood Pressure Intervention Trial [33]. Recently, associations for ePWV have also been connected to cognitive vascular aging, with ePWV being related to cognitive performance and the incidence of dementia in older adults [17, 18]. Although cognitive performance and vascular dementia are associated with worsened balance function [34–36], the connection between ePWV and balance function had yet to be explored. To our knowledge, the present study is the first to associate ePWV with a measure of physical function. We found that, even after adjustment for the constituent components of the ePWV equation and additional confounders, ePWV was significantly associated with the number of conditions passed on the MRT. These findings suggest that ePWV provides insight into the relationship between vascular aging and balance function. Additionally, we found that these significant associations were consistent in both males and females. Our observations of significant associations between ePWV and balance function across genders, as well as the practicality of ePWV due to only requiring age and BP, suggests that ePWV may be a useful clinical tool for general practitioners to identify men and women with poor balance control who may be at a high risk for falls and fall-related injuries.

### Limitations

Due to the nature of population datasets, unbalanced samples (i.e., for age, race) may limit the generalizability of our findings. The cross-sectional study design means that causality and temporality between ePWV and balance function cannot be inferred from these findings. NHANES also uses questionnaire data for the sociodemographic and physical activity variables, subjecting our findings to recall and response bias. Finally, although we did not use the gold standard measure of arterial stiffness (cfPWV), ePWV has been shown to significantly predict all-cause mortality, major CVD events, and cerebrovascular events independently of traditional CVD risk factors (including cfPWV) [15, 19, 20, 32] and may be a more appropriate and practical measure for providing insight into vascular aging in large, epidemiological studies due to only requiring age and BP.

### Conclusion

In conclusion, we found that increased ePWV is associated with worsened balance control in US adult men and women. As ePWV only requires age and BP, measures that are regularly taken during routine physical examinations, it may be an accessible and practical tool for general practitioners to identify individuals at a high risk for balance disorders, falls, and fall-related injuries. Future research should explore ePWV as a potential target for interventions aimed at improving balance control and lowering fall risk among US adults.

## Summary

### What is known about this topic?

- Vascular dysfunction has been shown to negatively impact indices of physical function, such as gait speed and muscular power, in adults.
- Recent studies have supported a link between arterial stiffness and balance function, with higher carotid-femoral pulse wave velocity associated with worsened postural control in older adults and higher postural sway in younger adults.
- Estimated pulse wave velocity (ePWV) is an independent predictor for all-cause mortality and clinical CVD outcomes, and is associated with worsening cognitive performance and dementia.

### What this study adds

- This study found that higher ePWV is associated with worse balance control in US adult men and women, suggesting ePWV may provide novel insight into the relationship between vascular aging and balance function.
- The present study adds to existing literature by suggesting that the clinical utility of ePWV extends beyond CVD outcomes, potentially providing valuable insight into physical functioning.

## Supplementary information

Table S1. General Linear Model

Table S2. Sex-Specific Analysis

## Data Availability

The data for this study and the descriptions for the study procedures are publicly available online at https://wwwn.cdc.gov/nchs/nhanes/continuousnhanes/default.aspx?Cycle=2021-2023.
