# Supplementary material for: The association between estimated pulse wave velocity and balance function in U.S. adults
Source: J Hum Hypertens. 2025 Sep 3;39(11):764–9. doi: 10.1038/s41371-025-01062-0 (PMC12592208; doi:10.1038/s41371-025-01062-0)
Supplement: Supplementary file 1 — Table S1. General Linear Model [file 41371_2025_1062_MOESM1_ESM.docx]

**Table S1. General Linear Model**

|  | **Estimate** | ***t*** | **95% CI** | ***p*-value** |
| --- | --- | --- | --- | --- |
| Gender (ref. female) | 0.018 | 0.545 | -0.054 – 0.091 | 0.594 |
| Race/Ethnicity |  |  |  |  |
| Mexican American | 0.069 | 0.793 | -0.116 – 0.255 | 0.440 |
| Other Hispanic | 0.006 | 0.060 | -0.197 – 0.209 | 0.953 |
| NH White | 0.031 | 0.361 | -0.150 – 0.211 | 0.723 |
| NH Black | 0.122 | 1.442 | -0.058 – 0.302 | 0.170 |
| NH Asian | 0.152 | 1.616 | -0.048 – 0.353 | 0.127 |
| Other race/multiracial (ref) | --- | --- | --- | --- |
| Educational Attainment |  |  |  |  |
| <9^th^ grade | -0.335 | -2.472 | -0.623 – -0.046 | *0.026 |
| 9^th^ – 11^th^ grade | -0.302 | -3.213 | -0.503 – -0.102 | *0.006 |
| High school diploma | -0.184 | -2.989 | -0.315 – -0.053 | *0.009 |
| Some college or AA degree | -0.158 | -3.830 | -0.246 – -0.070 | *0.002 |
| College graduate or above (ref) | --- | --- | --- | --- |
| SES | 0.021 | 1.582 | 0.007 – 0.050 | 0.134 |
| Alcohol Consumption | -0.001 | -5.738 | -0.001 – -0.001 | *<0.001 |
| Smoking ref. (<100 cigarettes) | -0.334 | -3.317 | -0.548 – 0.119 | *0.005 |
| BMI | 0.007 | 1.721 | -0.002 – 0.015 | 0.106 |
| MVPA | 0.001 | -0.571 | 0.000 – 0.001 | 0.576 |
| ePWV | -0.148 | -10.482 | -0.179 – -0.118 | *<0.001 |
