# Supplementary material for: The association between estimated pulse wave velocity and balance function in U.S. adults
Source: J Hum Hypertens. 2025 Sep 3;39(11):764–9. doi: 10.1038/s41371-025-01062-0 (PMC12592208; doi:10.1038/s41371-025-01062-0)
Supplement: Supplementary file 2 — Table S2. Sex-Specific Analysis [file 41371_2025_1062_MOESM2_ESM.docx]

**Table S2. Sex-Specific Analysis**

|  | **Estimate** | ***t*** | **95% CI** | ***p*-value** |
| --- | --- | --- | --- | --- |
| **Males** | | | | |
| **Unadjusted Model** | | | | |
| ePWV | -0.121 | -5.830 | -0.166 – -0.077 | *<0.001 |
| **Adjusted Model** |  |  |  |  |
| Age | 0.013 | 1.613 | -0.004 – 0.031 | 0.128 |
| SBP | 0.015 | 3.891 | 0.007 – 0.023 | *0.001 |
| DBP | 0.009 | 2.406 | 0.001 – 0.017 | *0.029 |
| Race/Ethnicity |  |  |  |  |
| Mexican American | 0.090 | 0.542 | -0.263 – 0.442 | 0.596 |
| Other Hispanic | 0.126 | 0.851 | -0.189 – 0.441 | 0.408 |
| NH White | 0.081 | 0.709 | -0.163 – 0.326 | 0.489 |
| NH Black | 0.195 | 1.605 | -0.064 – 0.455 | 0.129 |
| NH Asian | 0.243 | 2.047 | -0.010 – 0.496 | 0.059 |
| Other race/multiracial (ref) | --- | --- | --- | --- |
| Educational Attainment |  |  |  |  |
| <9^th^ grade | -0.386 | -1.704 | -0.870 – 0.097 | 0.109 |
| 9^th^ – 11^th^ grade | -0.278 | -2.611 | -0.506 – -0.051 | *0.020 |
| High school diploma | -0.195 | -2.325 | -0.373 – -0.016 | *0.034 |
| Some college or AA degree | -0.276 | -5.172 | -0.390 – -0.162 | *<0.001 |
| College graduate or above (ref) | --- | --- | --- | --- |
| SES | 0.025 | 1.282 | -0.016 – 0.066 | 0.219 |
| Alcohol Consumption | -0.001 | -5.469 | -0.001 – -0.001 | *<0.001 |
| Smoking ref. (<100 cigarettes) | 0.034 | 0.611 | -0.085 – 0.154 | 0.219 |
| BMI | 0.011 | 2.240 | 0.001 – 0.022 | *0.041 |
| MVPA | 0.001 | -0.063 | 0.000 – 0.001 | 0.950 |
| ePWV | -0.350 | -3.599 | -0.557 – -0.143 | *0.003 |
| **Females** | | | | |
| **Unadjusted Model** | | | | |
| ePWV | -0.169 | -15.722 | -0.192 – -0.146 | *<0.001 |
| **Adjusted Model** |  |  |  |  |
| Age | -0.005 | -1.333 | -0.013 – 0.003 | 0.203 |
| SBP | 0.004 | 1.495 | -0.002 – 0.011 | 0.156 |
| DBP | 0.007 | 1.513 | -0.003 – 0.017 | 0.151 |
| Race/Ethnicity |  |  |  |  |
| Mexican American | 0.175 | 1.595 | -0.059 – 0.409 | 0.132 |
| Other Hispanic | -0.031 | -0.287 | -0.261 – 0.199 | 0.778 |
| NH White | 0.047 | 0.472 | -0.164 – 0.258 | 0.644 |
| NH Black | 0.078 | 0.586 | -0.206 – 0.363 | 0.566 |
| NH Asian | 0.058 | 0.565 | -0.161 – 0.277 | 0.580 |
| Other race/multiracial (ref) | --- | --- | --- | --- |
| Educational Attainment |  |  |  |  |
| <9^th^ grade | -0.386 | -2.776 | -0.682 – -0.090 | *0.014 |
| 9^th^ – 11^th^ grade | -0.297 | -2.059 | -0.604 – 0.011 | 0.057 |
| High school diploma | -0.156 | -2.701 | -0.279 – -0.033 | *0.016 |
| Some college or AA degree | -0.007 | -0.135 | -0.121 – 0.107 | 0.894 |
| College graduate or above (ref) | --- | --- | --- | --- |
| SES | 0.051 | 3.315 | 0.018 – 0.084 | *0.005 |
| Alcohol Consumption | 0.005 | 0.872 | -0.007 – 0.017 | 0.397 |
| Smoking ref. (<100 cigarettes) | -0.147 | -1.041 | -0.449 – 0.154 | 0.314 |
| BMI | -0.001 | 0.812 | -0.011 – 0.010 | 0.849 |
| MVPA | 0.001 | -0.509 | 0.000 – 0.001 | 0.618 |
| ePWV | -0.199 | -4.501 | -0.294 – -0.105 | *<0.001 |
